# Graduate Study on the Continent

**Published:** 1908-07-11

**Authors:** 


### HOSPITAL ADMINISTRATION.

### CONSTRUCTION AND ECONOMICS.

### GRADUATE STUDY ON THE CONTINENT.—GERMANY.

VII.—THE SMALL *VERSUS* THE LARGE CENTRE.

The student would do well to bear in mind that post-graduate study means something more than the mere taking of courses. Its object, as an ideal, is to broaden his purview and enlarge his mind. Possibly that ideal is not very well understood in Germany. The professors often suppose a large amount of ignorance on the part of their audience, and we have heard, on more than one occasion, expressions of dissatisfaction from graduate students who have been participants in some special course or some particular work. After the accounts of the advance of the post-graduate study movement in Germany, which we have given in preceding chapters, it is only right that we should point this out—this defect, if one may call it so, in the conception of the post-graduate student which the average German specialist has. The orthopædic professor, in some cases at least, imagines that foreigners who attend his class know nothing about the subject; very often they know more about it, in a general way, than he does himself. Thus we have heard a privat-docent discoursing on a particular deformity to a post-graduate student who has made that lesion a specialty and contributed excellent work to its elucidation. To one who knew the facts it was extremely amusing to watch the polite attention which this latter practitioner paid to the discourse of the young professor. Again, it is an annoying experience to find the work in one's own department, done in one's own country, absolutely unknown and unrecognised abroad, priority ascribed to this or that foreign professor in methods of treatment which we were familiar with as dressers. Let no one imagine that graduate study on the Continent is wholly and entirely without these little annoyances, but in admitting their existence and in patiently bearing with them, the student will prove that he is true to his ideal, which is to learn to know. To know not only what methods excel ours, what knowledge is better than ours, but equally what methods fail in comparison with ours, and how profound in many instances medical ignorance may be!

For the development and furtherance of this acquiring of knowledge, which constitutes the ideal of post-graduate study, many factors are necessary beyond the purely personal. To a certain extent one cannot rightly benefit by foreign methods without having studied their application in their native home or without knowing how they are worked under the conditions which originated them. For that reason the graduate student needs to visit continental centres, although he can obtain almost as much information as he wants by reading assiduously the very excellent descriptions which are given in the larger monographs emanating from continental clinics.

#### The Smaller Centres.

For the graduate who has the time and the means to make a somewhat extended trip abroad, the ideal course to pursue would be to work for a time at a large centre and then to do his specialty at a smaller one. The quiet calm of a little University town is highly conducive to success in original research, if one may judge from the amount of solidly good work that has emanated from these minor centres. The question of the place of study is one which must be settled by every graduate on individual consideration, but it is worth while strongly to emphasise the claims of the smaller centres. These have hitherto been neglected by the English and American graduate. At many of them no English-speaking medical student has attended for many years, and one finds on inquiry that this neglect is simply due to the fact that the advantages of study at such centres are not known to the average graduate who comes to the Continent. The larger centres attract—to a greater degree than their importance in some cases warrants—the mass of English-speaking graduates, simply because no one has yet put forward the claims of the smaller centres. The following summaries may be of use to those intending to make one or other centre their special work-place. Our thanks are due to many professors and medical colleagues for valuable information and advice which have been freely used in drawing up these notes.

Göttingen.—Of all the smaller German universities Göttingen should specially appeal to the English graduate, because it served for many years as an important educative centre for Englishmen. Here von Haller, Gmelin, Murray the botanist, and Richter, the disciple of Percival Pott and the first of German surgeons of his day, taught, and in surgery, at least, the Göttingen school has made history. Göttingen is a typical University town, and the social life of the community is greatly influenced by the University. In the Aussenstadt quarter or in the vicinity of the Albaniterthor, near the clinics, the student can obtain comfortable quarters, *en garçon*, at a very moderate rental. It is customary here (as indeed in most smaller centres) to hire rooms by the quarter, monthly contracts being rarely made. The average rental of a bed-sitting-room is 100 marks per semester; some quite comfortable rooms may be obtained for 80 marks per semester; larger ones run to 150 or 200 marks. This does not include breakfast nor heating, but "boots" and attendance are included. Breakfast can be arranged for at from 3 to 10 marks per month. There are excellent pensions at which the graduate may obtain board and lodging at the low rate of 100 marks per month. The requirements for matriculation are the same as in Berlin (see first article of this series). Indeed the details which have been already given with regard to matriculation at the Berlin University will serve for all other centres, with the exception of the Bavarian Universities, which will be dealt with later on.

As a centre for post-graduate study Göttingen appears to be excellent. Among its professors are Professors Cramer (psychiatry), Braun, and Rosenbach (surgery), Hirsch (medicine), and Ehrlich (pathology). There is an exceptionally good library, and a fine pathological and anatomical museum, rich in ethnographical specimens. The clinics are small, but offer good material, and the hospital is quite up-to-date. Courses are given during the vacation for "practising physicians"—usually free to all German practitioners. The foreign graduate needs special permission from the professor to attend these courses. Details of these courses, as in fact for all post-graduate courses in Germany, are published periodically in the Berlin Journal of the German Post-graduate Association. Many of the privat-docenten give private courses at fees varying from 15 to 60 marks per month. The courses given in medicine and pathology are very good.

The Northern Universities.—These are generally to be recommended only during the winter months. During the summer term few courses are given and little practical work is done. At each post-graduate courses are given during April and October. Greifswald, with 180 medical students, is one of the cheapest of German University towns. The fees are very low, and a large comfortable room may be rented at 60 to 80 marks for the whole semester. The Greifswald student is said to live comfortably on 80 marks a month, everything included. Pensions are few, but cheap and good. The professors take an individual interest in their students, and it is customary to invite the latter to the municipal and professional balls and parties. Among the professors are Professors Minkowski (medicine), Schultze (psychiatry), and Loeffler (hygiene and bacteriology). The hospital and clinics are small, and there is not much scope for surgical work. For medical, pathological, and bacteriological work this centre is excellent. Kiel is a comparatively large University centre with nearly a thousand students, three hundred of whom are medical. It is not a cheap centre, the "student's average" being fairly high—150 marks per month. In the Brunswick quarter, an old part of the town, close to the hospitals, a bed-sitting-room can be rented for 20 to 40 marks per month. Pension, which is by no means so good as at other centres, varies from 140 to 200 marks per month. Very little work is done during the summer semester. The hospitals are good, and there is scope for special work in ear, nose, and throat. Among the professors are Professors Petersen (surgery), Hoppe-Seyler (medicine), and Friedrich (otology). The climate is somewhat like that of Aberdeen. Many special and private courses are given during the winter semester, the fees being, on the whole, low. Königsberg, like Kiel, is not a cheap centre, but pensions are better, ranging from 100 to 160 marks per month. There are many social advantages, and foreign graduates may become members of the fine Palaestra Albertina, a club founded by Dr. Lange, of New York, for the students. Professors Stieda (surgery), Pfeiffer (hygiene), and Meyer (neurology) are on the staff, and there are many well-known privat-docenten. Clinical material is abundant at Königsberg, and there are many opportunities for special work. A very large number of post-graduate courses may be attended during the summer vacation. These are very popular with Russian practitioners, who state that they are all good, and that practical work is especially considered. For the most part such courses are free, a nominal fee being paid for registration. Rostock is a much smaller centre, with only 130 medical students. Rooms vary from 20 to 40 marks per month, and living is cheaper than at Kiel or Königsberg. At this centre Professor Thiesfelder gives special classes in pathological anatomy, and Professor Müller in surgery, and in both subjects there are exceptionally good opportunities for study. The hospitals are small, and an assistantship is difficult to obtain, but private courses are given. The centre deserves to be better known to foreign graduates than it is at present.

Hamburg, Bremen and Cologne.—None of these are University towns, but each of them possesses very fine hospitals at which much good work can be done by the graduate student. Living is comparatively cheap, except at Hamburg, where it is about the same as at Berlin. Post-graduate courses are given at all the hospitals during October and April, but are not open to foreign graduates. A letter to the professor is usually sufficient to obtain permission to attend, and the courses are exceptionally good. No graduate student who is interested in general surgery should fail to see the excellent work done at Professor Kümmel's clinic at Hamburg-Eppendorf. The amount of clinical material is enormous, and the facilities offered for special investigation and in studying particular subjects are such as to make these cities equal to the larger medical centres so far as the foreign graduate is concerned. We would specially draw attention to the invariable courtesy and kindliness which are shown to foreign visitors at the large Hamburg Hospitals. At Eppendorf the foreign graduate is treated with the utmost consideration, and, whether he takes a course or not, is allowed to witness operations, attend the physicians, and to make use of the fine hospital, undoubtedly the best on the Continent, as much as he pleases. Hamburg especially deserves to be visited by the post-graduate student, especially by those who are interested in surgery, because of the excellent post-graduate courses which are given at its city hospitals during April and October.

Heidelberg.—This centre is so well known that it needs no more than a brief notice here. The names of Professors Erb (medicine), Nissl (neurology), Narath (surgery), Kümmel (otology), and v. Krehl (medicine) are known outside Germany, and besides these many others of a more than local reputation appear on the list of the staff. There are 250 medical students, and the hospitals, laboratories, and private clinics are very good. In physiological, pathological, and surgical work the graduate student will find many opportunities for study. An unusually full list of post-graduate courses at this centre is published at the commencement of every vacation. Living is cheap, and the pensions are good. Private courses are somewhat more expensive here than at other small centres. For research work there are few places that are so attractive. The fine old library, and the equally fine collections attached to the various laboratories, are available for students, and the clinics offer material which is as good as any that can be obtained in a small German University centre. The English colony at Heidelberg is a fairly influential and large one, and most of the professors speak English fluently. Here, as elsewhere, however, the student cannot expect to do much unless he makes up his mind to master German. As we have already stated, the acquisition of a working knowledge of the language is by no means very difficult, and will prove of the greatest use afterwards. Even in cases where courses of lectures are professedly given in English, the student will find that a knowledge of German is of great value. The English lecture given by the German professor is in the majority of cases a studied harangue, and one cannot reasonably expect it to be as good as the lecture which would have been delivered in German had the lecturer felt that his audience would have been able to understand him as well in his native tongue.

Halle can hardly be classed as a small centre, yet it may be taken here. Of late years it has steadily added to its reputation as an excellent centre for medical study. The best known of its professors are Professor Eberth (pathological anatomy), Fränkel (hygiene), Schwarz (gynæcology), Frommer (midwifery), and Stieda (surgery). Very many post-graduate courses are given here during the vacation. Cost of living at Halle is somewhat higher than at most German universities, varying from 150 to 200 marks per month, but the choice of pensions is almost unlimited. Very good opportunities are offered to the student for the study of special subjects such as pathology, bacteriology, gynæcology, and pure medicine. Halle has a good many foreign students, and among its medical students are both English and American graduates.

